# Fabrication and Electrical Characterization of MgZnO/ZTO Thin-Film Transistors

**DOI:** 10.3390/nano15231809

**Published:** 2025-11-29

**Authors:** Yunpeng Hao, Chao Wang, Liang Guo, Yu Sun, Meihua Jin, Linbo Xu, Ying Huang, Yi Zong, Xiwen Xu, Jingxuan Zeng

**Affiliations:** 1 Department of Equipment Manufacturing and Intelligent Control, Yanbian Vocational & Technical College, Yanji 133000, China; apeng0031@163.com (Y.H.); 18643328644@163.com (Y.S.); 18643398998@163.com (M.J.); xiaoguaixlb@126.com (L.X.); 13843527885@163.com (Y.H.); 13179093335@163.com (Y.Z.); 18744433976@163.com (X.X.); 18843300339@163.com (J.Z.); 2 Key Laboratory of Architectural Cold Climate Energy Management, Ministry of Education, Jilin Jianzhu University, Changchun 130118, China; guoliang@jlju.edu.cn; 3 Jilin Provincial Key Laboratory of Architectural Electricity & Comprehensive Energy Saving, Jilin Jianzhu University, Changchun 130118, China

**Keywords:** MgZnO/ZTO-TFTs, XPS, electrical performance, stability

## Abstract

To enhance the electrical performance of MgZnO-TFTs, this study employed radio-frequency (RF) magnetron sputtering to fabricate MgZnO/ZTO thin films. Using these films as the channel layer, bottom-gate top-contact MgZnO/ZTO-TFT devices were constructed. The thin films were characterized using atomic force microscopy (AFM) and X-ray photoelectron spectroscopy (XPS). After optimization, the MgZnO/ZTO-TFT exhibited a high field-effect mobility of 16.80 cm^2^·V^−1^·s^−1^, high Ion/off of 7.63 × 10^8^, threshold voltage of −1.60 V, and subthreshold swing as low as 0.74 V·dec^−1^. Bias stress stability tests were conducted under positive bias stress (PBS) and negative bias stress (NBS) conditions with a source-drain voltage of 20 V and gate bias stresses (VGS) of +10 V and −10 V, respectively, for a duration of 1000 s. The resulting threshold voltage shifts were only +0.58 V and −0.15 V, respectively, indicating excellent bias stability. These results suggest that the ZTO film, serving as the lower channel layer, effectively enhances carrier transport at the MgZnO/ZTO interface, thereby improving the field-effect mobility and on/off current ratio. Meanwhile, the MgZnO film as the upper channel layer adjusts the device’s threshold voltage and enhances its bias stability.

## 1. Introduction

In recent years, zinc oxide (ZnO) has gained significant attention as a third-generation semiconductor material owing to its excellent electrical properties. Utilizing ZnO-based active layers in thin-film transistors (TFTs) allows devices to achieve superior electrical performance. However, intrinsic defects in ZnO, such as oxygen vacancies and zinc interstitials, significantly degrade TFT performance [1].

Based on ZnO semiconductor materials, doping ZnO with metal elements such as In [2], Ga [3], Sn [4], and Mg [5] to prepare the active layer exerts varying effects on the performance of TFTs devices. Owing to its exceptional performance, IGZO-TFT has emerged as the core upgrade path from conventional amorphous silicon (a-Si) technology, establishing a strong competitive position in specific applications such as high-end televisions and professional monitors [6,7]. However, the In and Ga used in IGZO films are rare metals and are expensive. Moreover, In poses potential toxicity issues. These drawbacks somewhat constrain the sustainable development of the industry. Therefore, researchers are actively exploring alternative elements to reduce reliance on these two metals. In recent years, multiple studies have shown that doping zinc oxide with Mg to form MgZnO films and using them as the active layer in TFTs can, to some extent, improve the electrical performance of zinc oxide-based TFT devices. This improvement is attributed to the similar ionic radii of magnesium and zinc ions (Mg^2+^: 0.57 Å, Zn^2+^: 0.60 Å), which minimizes significant lattice distortion upon Mg doping. Additionally, introducing Mg shifts the conduction band edge upward and may distance it from inherent shallow donor states, thereby increasing the activation energy of defect donors [8,9]. However, Mg doping also affects the field-effect mobility of the devices. In previous studies, MgZnO-TFTs exhibited a carrier mobility of only 0.29 cm^2^·V^−1^·s^−1^ and an on/off current ratio of 1.68 × 10^6^ [10]. The underlying reasons are hypothesized to include grain boundary scattering caused by reduced grain size, impurity scattering resulting from higher Mg concentrations, and widened bandgaps leading to an increased electron effective mass [11]. Nevertheless, TFTs with MgZnO active layers demonstrate better stability compared to those with undoped ZnO active layers [12,13].

To enhance the performance of MgZnO-TFTs, numerous researchers have adopted a dual-channel layer structure by introducing a ZnO film beneath the MgZnO layer, As shown in Table 1. For instance, Jong Hoon Lee et al. [14] fabricated polycrystalline MgZnO/ZnO-TFTs on thermally oxidized silicon substrates using radio-frequency (RF) magnetron sputtering and achieved a field-effect mobility of 7.56 cm^2^·V^−1^·s^−1^. Similarly, Dewu Yue et al. [15] optimized the film synthesis conditions and produced MgZnO/ZnO-TFTs with a field-effect mobility of 21.1 cm^2^·V^−1^·s^−1^ and an Ion/off of 1.5 × 10^8^. Although MgZnO/ZnO structures demonstrate potential for practical applications, previous studies have indicated that improvements in field-effect mobility or on/off current ratio often come at the expense of the device’s bias stability.

**Figure 1:**
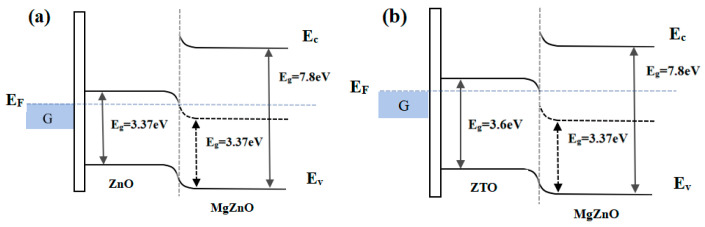
Schematic diagram of the band gap (**a**) ZnO and MgZnO (**b**) ZTO and MgZnO.

According to previous reports, ZTO-TFTs exhibit superior electrical performance and enhanced bias stress stability compared to ZnO-TFTs [16,17]. This improvement can be attributed to the broader bandgap of ZTO material (E_g_ = 3.6 eV at room temperature) [18] relative to ZnO (E_g_ = 3.37 eV at room temperature) [19]. When ZTO is combined with MgZnO—whose bandgap is continuously tunable from 3.37 eV to 7.8 eV [20] based on the Mg doping ratio—to form a dual-active-layer device, the energy barrier during operation is reduced, leading to higher carrier transport rates and more efficient injection [21], as shown in Figure 1. To enhance electrical performance while maintaining good bias stability, this study selects ZTO thin film as the bottom channel layer. A MgZnO/ZTO-TFTs was fabricated using magnetron sputtering, and the influence of ZTO film growth conditions on the electrical characteristics of the MgZnO/ZTO-TFT was investigated, along with an evaluation of its bias stress stability.

## 2. Experiment Section

**Figure 2:**
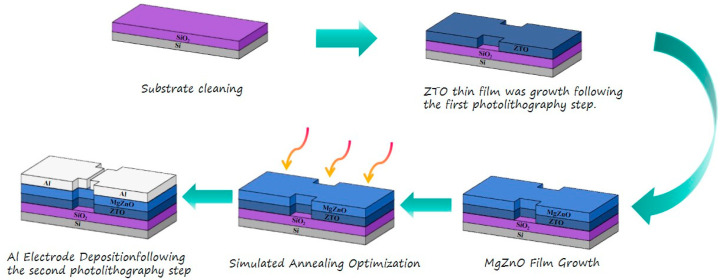
Fabrication process flow of MgZnO/ZTO-TFTs.

This experiment primarily employed the magnetron sputtering method for device fabrication. p-Si substrates were selected for device construction, and a silicon dioxide insulating layer with a thickness of 285 nm was prepared via thermal oxidation. Initially, the substrates were ultrasonically cleaned in acetone, ethanol, and deionized water to remove surface contaminants and mitigate the impact of surface defects on device performance. Following the first photolithography step to pattern the active layer, ZTO thin films were deposited at room temperature using a PVD75 Magnetron Sputtering System Kurt J. Lesker, USA. A 99.99% ZTO target with a ZnO:SnO_2_ ratio of 7:3 was used, with a sputtering power of 100 W and a sputtering pressure of 8 Torr. The argon-to-oxygen ratio and sputtering time were adjusted accordingly. Subsequently, MgZnO thin films were prepared by co-sputtering deposition at room temperature using 99.99% ZnO and Mg targets. The sputtering powers for the ZnO and Mg targets were set at 80 W and 15 W, respectively, with an argon-to-oxygen ratio of 85%:15% and a sputtering pressure of 8 Torr during the process. The thickness of the active layer was 30 nm. After the deposition of the active layer, the samples were annealed in an air atmosphere at 600 °C for 1 h using a German-made RTP-100 rapid thermal processor to optimize their properties. Following the second photolithography step to define the channel layer, 50 nm-thick Al electrodes were deposited using an China Taiwan Liangjie Technology Company electron beam evaporation equipment, completing the device fabrication. The channel length and width were 300 μm and 10 μm, respectively. The device fabrication process is illustrated in Figure 2 below.

## 3. Effect of Sputtering Ar:O_2_ Ratio in the Bottom Channel Layer on Device Performance

### 3.1. Surface Morphology Analysis of Double-Active-Layer Thin Films

**Figure 3:**
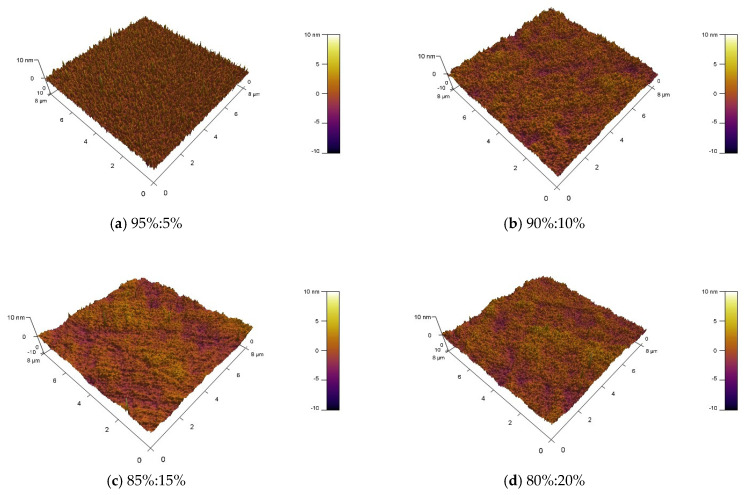
AFM images of MgZnO/ZTO films deposited at Ar/O_2_ ratios of (**a**) 95%:5% (**b**) 90%:10% (**c**) 85%:15% (**d**) 80%:20%.

The surface roughness of the thin film was analyzed using a MFP-3D Origin+ Atomic Force Microscope produced by Oxford Instruments with scanning performed over an 8 μm^2^ area, as shown in Figure 3. The overall root-mean-square (RMS) roughness of the ZTO film was measured as follows: 0.895 nm at an argon-to-oxygen ratio of 95%:5%; 0.842 nm at 90%:10%; 0.88 nm at 85%:15%; and 0.913 nm at 80%:20%. This indicates that the overall surface roughness initially decreased and then increased, remaining below 1 nm in all cases. The lowest surface roughness of 0.84 nm was observed at an argon-to-oxygen ratio of 90:10. This phenomenon can be attributed to the following: At an oxygen flow rate of 5%, the excessively low oxygen content resulted in increased defects in the underlying active layer, thereby elevating the overall surface roughness. When the oxygen flow rate exceeded 10%, excess oxygen led to an increase in grain size within the film, subsequently increasing the overall surface roughness [22,23].

**Figure 4:**
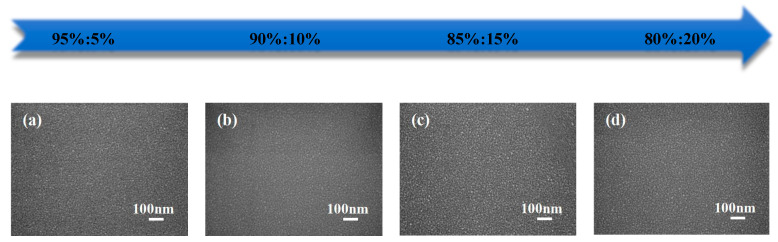
SEM images of ZTO films deposited at various Ar/O_2_ sputtering ratios (**a**) 95%:5% (**b**) 90%:10% (**c**) 85%:15% (**d**) 80%:20%.

Figure 4 shows representative SEM images of the ZTO film deposited under different sputtering argon-to-oxygen ratios. Consistent with the AFM analysis, the surface was rougher at an oxygen flow rate of 5%. Upon increasing the oxygen flow rate to 10%, the film surface exhibited finer and more uniform grains. However, with further increases in the oxygen flow rate, the grain size on the film surface increased, accompanied by the formation of microscopic voids.

### 3.2. XPS Analysis of ZTO Thin Films

**Figure 5:**
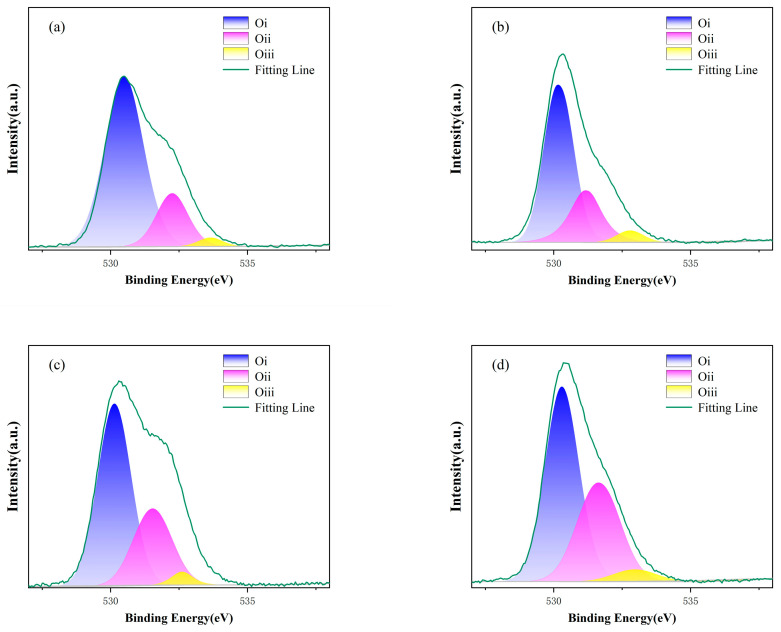
The XPS spectra of ZTO thin films in (**a**) 95%:5% (**b**) 90%:10% (**c**) 85%:15% (**d**) 80%:20% sputtered argon-oxygen ratios.

Figure 5 presents the O-1s XPS spectra of ZTO thin films annealed under different atmospheres. The spectra reveal three distinct chemical states: Oi (530.3 ± 0.2 eV), Oii (531.4 ± 0.25 eV), and Oiii (532.8 ± 0.2 eV). Oi corresponds to lattice oxygen, Oii represents the oxygen vacancy (Vo) concentration within the film, and Oiii is attributed to surface-adsorbed oxygen-containing species, such as hydroxyl groups (-OH). The relative proportions of Oi, Oii, and Oiii derived from the O-1s spectra are summarized in Table 2. Under the four annealing atmospheres investigated, the proportion of Oi was 77.6%, 69.1%, 65.3%, and 58.6%, respectively. This indicates that during annealing, the majority of oxygen atoms bonded with metal atoms to form metal oxides, exhibiting high binding energy. As the oxygen flow rate increased during annealing, the V_o_ content in the films progressively increased. However, excessive V_o_ concentrations can introduce detrimental defects, degrading device performance [24].

### 3.3. Analysis of Electrical Properties

**Figure 6:**
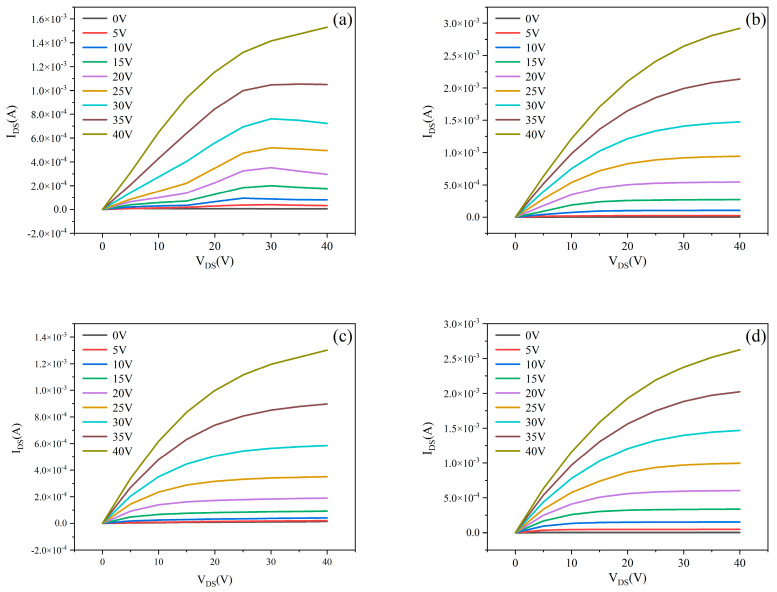
The output curve of MgZnO/ZTO-TFTs at (**a**) 95%:5% (**b**) 90%:10% (**c**) 85%:15% (**d**) 80%:20% sputtered argon-oxygen ratios.

The electrical performance of MgZnO/ZTO-TFT devices—fabricated by modulating the sputtering Ar/O_2_ ratio during ZTO thin-film deposition—was characterized using a semiconductor parameter analyzer. For output characteristics, the gate voltage (V_GS_) was scanned from 0 to 40 V in 5 V increments, while the V_DS_ was swept from 0 to 40 V. Figure 6 displays the output characteristic curves of MgZnO/ZTO-TFTs prepared with varying ZTO sputtering Ar/O_2_ ratios. As observed, the drain-source current (I_DS_) scales proportionally with increasing V_GS_ across all devices. At V_GS_ = 40 V, all four samples achieved currents in the milliampere range (10^−3^ A).

**Figure 7:**
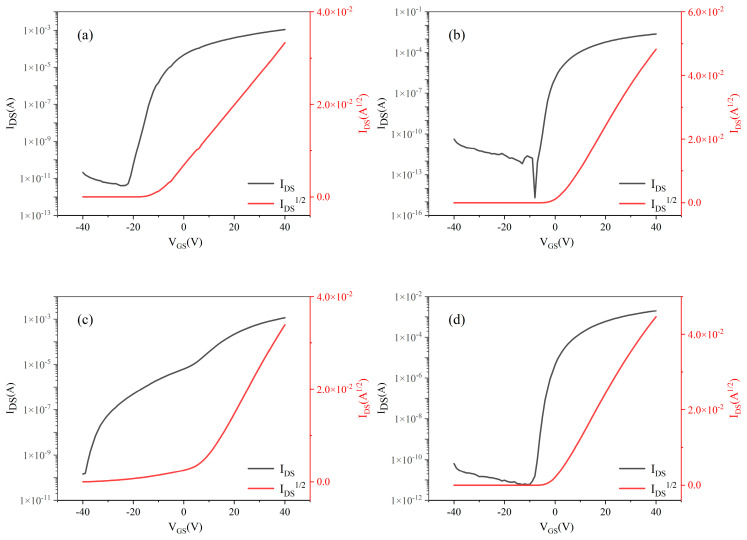
The transfert curve of MgZnO/ZTO-TFTs at (**a**) 95%:5% (**b**) 90%:10% (**c**) 85%:15% (**d**) 80%:20% puttered argon-oxygen ratios.

Figure 7 displays the transfer characteristics of MgZnO/ZTO-TFTs fabricated with ZTO thin films deposited at varying sputtering Ar/O_2_ ratios. With the drain-source voltage fixed at 20 V, the gate voltage was scanned from −40 to 40 V. As observed, all devices exhibit well-defined switching characteristics across the tested Ar/O_2_ ratios.

Table 2 summarizes the electrical performance parameters of MgZnO/ZTO-TFTs fabricated using ZTO thin films deposited at varying sputtering Ar/O_2_ ratios. The field-effect mobility (μ_sat_), subthreshold swing (SS), and current Ion/off were derived from Equations (1)–(3).

(1) $$\begin{array}{c} \mu_{sat}={\frac{2L}{WC_{i}}\left(\frac{\partial \sqrt{I_{DS}}}{\partial V_{GS}}\right)}^{2}\; \end{array}$$

(2) $$\begin{array}{c} SS=\frac{dV_{GS}}{d\left(\log I_{DS}\right)} \end{array}$$

(3) $$\begin{array}{c} \frac{I_{on}}{I_{off}}=\frac{{\left(I_{DS}\right)}_{max}}{{\left(I_{DS}\right)}_{min}} \end{array}$$

Here, I_DS_ denotes the current between the source and drain; V_GS_ denotes the voltage between the gate and source, and L and W are the length and width of the device channel, respectively, C_i_ is the unit capacitance of the gate insulator, I_DS_(max) is the maximum drain-source current, and I_DS_(min) is the minimum drain-source current.

Table 3 presents the electrical performance parameters of MgZnO/ZTO-TFTs fabricated using ZTO thin films deposited under different sputtering argon-oxygen ratios. As shown in the table, the field-effect mobility of the experimentally prepared MgZnO/ZTO-TFTs shows significant improvement over that of MgZnO-TFTs. This enhancement stems from the dual-active-layer structure, which separates the carrier generation layer (with high defect density) from the highly conductive layer (with low defect density). This separation improves mobility and helps reduce the subthreshold swing [25]. The subthreshold swings measured for samples prepared under all four conditions are relatively small, indicating effective gate control over the devices. Specifically, for the ZTO film sputtered with an Ar:O_2_ ratio of 90%:10%, the subthreshold swing is only 0.02 V/dec, and the field-effect mobility reaches 10.46 cm^2^·V^−1^·s^−1^.Regarding the current on/off ratio, the on-state currents for all four samples are on the order of 10^−3^ A, while the off-state currents remain below 10^−10^ A. Notably, for the device with the ZTO film deposited at an Ar:O_2_ ratio of 90%:10%, the off-state current decreases to the order of 10^−13^ A, achieving an on/off current ratio of 2.69 × 10^9^, which demonstrates excellent switching performance. However, device performance deteriorates as the oxygen flow rate increases during sputtering. This occurs because the oxygen vacancy concentration in the ZTO film varies with the oxygen flow rate, affecting charge transport between the active layers. Increased oxygen flow rate raises the oxygen vacancy concentration, leading to oxygen ions escaping from the lattice which hinders charge propagation in the lower active layer and consequently reduces the overall carrier mobility of the device [26]. Additionally, MgZnO/ZTO-TFTs fabricated with sputtering Ar:O_2_ ratios of 95%:5% and 80%:20% exhibit depletion-mode characteristics. Both excessive and insufficient oxygen vacancy concentrations result in a pronounced negative shift in threshold voltage compared to the other samples. Based on the above analysis, the MgZnO/ZTO-TFT fabricated with a ZTO film deposited at a sputtering Ar:O_2_ ratio of 90%:10% demonstrates optimal performance, characterized by: field-effect mobility of 10.46 cm^2^·V^−1^·s^−1^, threshold voltage of 2.44 V, subthreshold swing of 0.02 V/dec, Ion/off of 2.69 × 10^9^,on-state current of 2.33 × 10^−3^ A, ultralow off-state current of 8.66 × 10^−13^ A.

## 4. The Effect of ZTO Thin Film Thickness on the Electrical Properties of MgZnO/ZTO-TFT

### 4.1. Surface Morphology Analysis of Double-Active-Layer Thin Films

**Figure 8:**
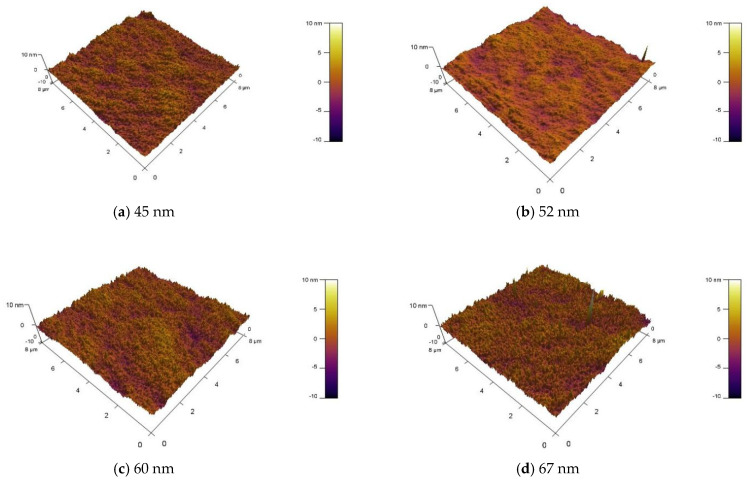
AFM images of MgZnO/ZTO films at (**a**) 45 nm (**b**) 52 nm (**c**) 60 nm (**d**) 67 nm.

Figure 8 presents atomic force microscopy (AFM) analysis of the surface morphology of MgZnO/ZTO thin films prepared under different sputtering argon-oxygen ratios. For a ZTO film thickness of 45 nm, the root-mean-square (RMS) roughness of the MgZnO/ZTO film, scanned over an 8 μm^2^ area, is 0.84 nm. At thicknesses of 52 nm, 60 nm, and 67 nm, the RMS roughness decreases to 0.75 nm, increases to 0.97 nm, and further increases to 1.02 nm, respectively. The variation in the thickness of the underlying active layer influences the surface roughness of the MgZnO/ZTO film. As the thickness increases, the overall RMS roughness initially decreases and then increases, reaching a minimum value of 0.75 nm at a thickness of 52 nm. This relatively low surface roughness satisfies the requirements for large-area device fabrication.

### 4.2. Analysis of Electrical Properties

**Figure 9:**
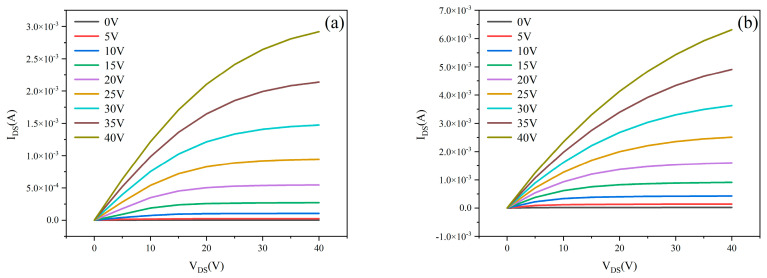

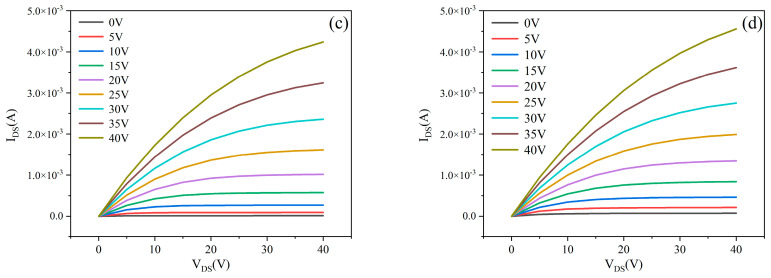
The toutput curve of MgZnO/ZTO-TFTs at ZTO film thickness (**a**) 45 nm (**b**) 52 nm. (**c**) 60 nm (**d**) 67 nm.

The Figure 9 Vbelow shows the output characteristic curves of MgZnO/ZTO-TFTs fabricated with ZTO films of varying thicknesses, scanned under the same conditions as described above. At V_GS_ = 40 V, the saturation current of MgZnO/ZTO-TFTs consistently remains on the order of 10^−3^ A as the ZTO thickness increases. Notably, for a 52 nm ZTO film, I_DS_ sat reaches 6.31 × 10^−3^ A.

**Figure 10:**
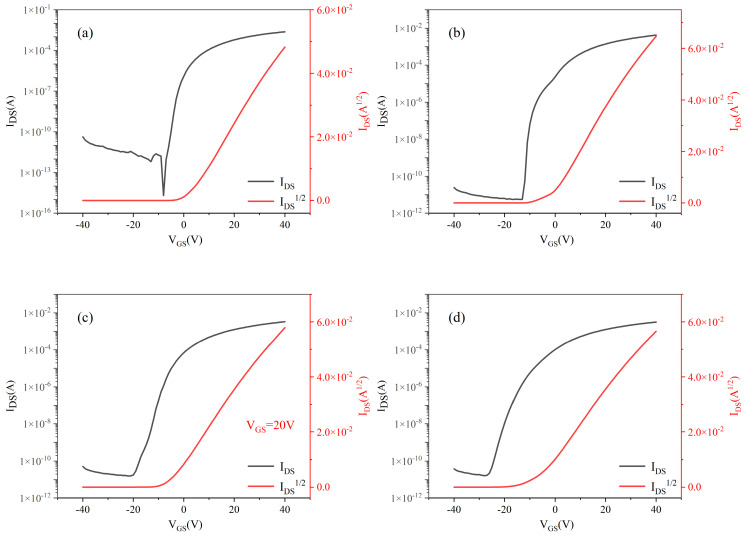
The ransfert curve of MgZnO/ZTO-TFTs at ZTO film thickness (**a**) 45 nm (**b**) 52 nm. (**c**) 60 nm (**d**) 67 nm.

Figure 10 displays the transfer characteristics of MgZnO/ZTO-TFTs annealed at different thickness. Measurements were performed with V_DS_ of 20 V, while V_GS_ was scanned from −40 V to 40 V. As shown, all devices—regardless of ZTO film thickness—exhibit excellent switching performance.

Table 4 shows the electrical performance parameters of MgZnO/ZTO-TFTs fabricated with different thicknesses of ZTO thin films. The data in the table indicate that varying the thickness of the ZTO layer significantly affects the device performance. Under all four conditions, the fabricated MgZnO/ZTO-TFTs still exhibit strong gate modulation capability. The change in ZTO film thickness has a considerable impact on the carrier mobility of the devices. This is because, as the thickness increases, the concentration of oxygen vacancies in the ZTO film also changes. When the oxygen vacancies continue to increase, the total density of defect states in the bottom active layer rises, leading to an increase in the number of carrier-trapping regions. This results in a reduction of carriers in the bottom active layer, degrading its conductivity and limiting the supply of sufficient free electrons to the top active layer [27,28]. Furthermore, when the lower active layer exhibits high conductivity, the elevated carrier concentration at the dual-active-layer interface effectively screens grain boundary potential barriers, reducing carrier scattering. Consequently, carrier transport efficiency improves, significantly enhancing field-effect mobility [29,30]. Thus, mobility initially increases but subsequently decreases with increasing lower active layer thickness. The Ion/off current ratio also varies with lower active layer thickness during switching operations. Under negative gate bias, carriers accumulate predominantly at the interface between the two active layers. In contrast, positive gate bias induces carrier accumulation both at the inter-layer interface and near the lower active layer/insulator interface. For the MgZnO/ZTO-TFTs with a 52 nm ZTO layer, the channel’s low turn-on voltage enables substantial carrier accumulation under maximum positive bias, increasing the on-state current. However, under negative bias, carrier accumulation near the upper/lower active layer interface creates a low-resistance path, elevating off-state leakage current [27,31]. Compared to the 45-nm device, the 52-nm TFT exhibits a marginally lower Ion/off yet maintains a ratio >10^8^. Collectively, the MgZnO/ZTO-TFT with a 52 nm ZTO layer delivers optimal performance: field-effect mobility of 16.80 cm^2^·V^−1^·s^−1^, threshold voltage of −1.60 V, subthreshold swing of 0.74 V/dec, Ion/off of 7.63 × 10^8^, on-state current of 4.18 mA and ultralow off-state current of 5.48 × 10^−12^ A.

## 5. Stability Study of MgZnO/ZTO-TFTs

**Figure 11:**
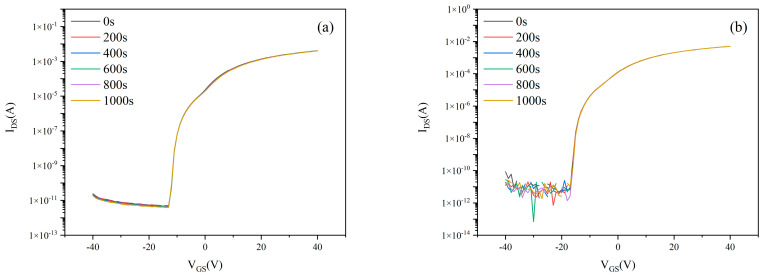
Transfer characteristics of MgZnO/ZTO-TFTs under different stress durations at gate bias stress of (**a**) +10 V (**b**) −10 V.

**Figure 12:**
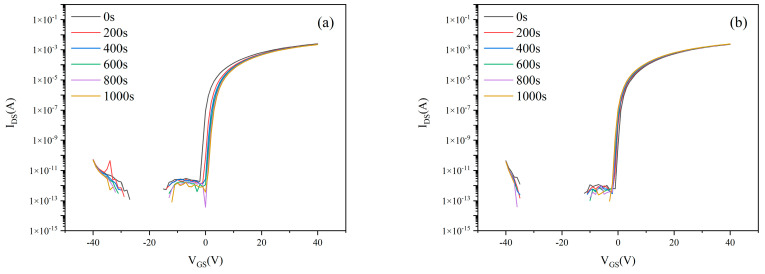
Transfer Characteristics of Conventionally Fabricated ZTO-TFTs under Different Stress Durations at gate bias stress of (**a**) +10 V (**b**) −10 V.

The preceding section optimized the growth conditions of the lower active layer, culminating in peak performance for MgZnO/ZTO-TFTs fabricated under specific parameters: ZTO sputtering power of 100 W, Ar:O_2_ ratio of 90%:10%, and thickness of 52 nm—all tested after 180-day ambient exposure without encapsulation. This subsection analyzes the bias stability of MgZnO/ZTO-TFTs prepared under these optimal conditions. Figure 11 show transfer characteristics under varying stress durations with V_DS_ = 20 V and gate biases of +10 V and –10 V. Table 5 below shows the stability data, after 1000 s of stress, threshold voltage shifts (ΔV_TH_) was: +0.58 V at V_GS_ = +10, –0.15 V at V_GS_ = −10 V. For comparison, conventionally fabricated ZTO-TFTs underwent identical bias stress testing. Figure 12 present their transfer characteristics under equivalent conditions. After 1000 s stress, ΔV_TH_ was +4.38 V at V_GS_ = +10 V, –3.32 V at V_GS_ = −10 V.

Electrical testing confirmed that under +10 V gate bias stress for 1000 s, the ΔVth of MgZnO/ZTO-TFTs was reduced to 0.58 V, significantly lower than the 4.38 V shift observed in conventional ZTO-TFTs. Similarly, under −10 V bias, ΔVth decreased from −3.32 V to −0.15 V, demonstrating superior bias stability. The relatively poor bias stability of ZTO-TFT may be attributed to defects in the deep gap states between the insulating layer and the semiconductor layer [4]. During current transport, these defects extensively trap free electrons, leading to a significant shift in the threshold voltage [32]. Under bias conditions, when a drain-source current is applied to the device, the current preferentially flows through the MgZnO/ZTO interface during transmission, rather than the ZTO/SiO_2_ interface. The formation of the MgZnO/ZnO heterojunction can generate a higher electron density at the interface, reducing interface scattering and thereby creating a more stable electron transport path [14,33]. Furthermore, the MgZnO upper active layer suppresses interface trap state density, minimizing charge trapping under bias stress and thereby improving stability [34,35].

## 6. Conclusions

In summary, we have successfully fabricated MgZnO/ZTO-TFTs exhibiting excellent performance. While significantly enhancing the electrical properties of the MgZnO-TFTs, the devices maintained excellent bias stability. This improvement is attributed to the bilayer structure: the ZTO film serves as the lower active layer, supplying sufficient carriers, while the MgZnO film acts as the upper active layer, establishing a more stable electron transport pathway through its polarization effects. The fabricated devices achieved a field-effect mobility of 16.80 cm^2^·V^−1^·s^−1^ and Ion/off up to 10^8^. Under a gate bias stress of VGS = ±10 V for 1000 s, the threshold voltage exhibited minimal shifts of +0.58 V and −0.15 V, respectively. Our work provides valuable insights for the subsequent development of high-performance TFTs devices.

## Figures and Tables

**Table 1:** Performance of MgZnO-Based Thin-Film Transistors.

	μ_sat_ (cm^2^ V^−1^ s^−1^)	V_TH_ (V)	SS (V/dec)	I_on_/I_off_
MgZnO-TFTs [10]	0.3	2.28	3.60	1.68 × 10^6^
MgZnO/ZnO-TFTs [14]	7.56	3.1	0.86	10^5^
MgZnO/ZnO-TFTs [15]	21.1	--	--	1.5 × 10^8^

**Table 2:** Composition of O-1s XPS profile of ZTO film in different sputtered argon-oxygen ratios.

Sputtering Ar/O_2_ Ratio	Oi	Oii	Oiii
95%:5%	74.57%	22.42%	2.83%
90%:10%	69.08%	26.03%	4.89%
85%:15%	65.25%	31.38%	3.37%
80%:20%	58.89%	36.61%	4.51%

**Table 3:** Electrical performance parameters of MgZnO/ZTO-TFT prepared by ZTO filmnder different sputtering argon-oxygen ratios.

Sputtering Ar/O_2_ Ratio	μ_sat_ (cm^2^·V^−1^·s^−1^)	V_TH_ (V)	SS (V/dec)	I_on_	I_off_	I_on_/I_off_
95%:5%	3.26	−7.60	1.74	1.11 × 10^−3^	4.07 × 10^−12^	2.73 × 10^8^
90%:10%	10.46	2.44	0.018	2.33 × 10^−3^	8.66 × 10^−13^	2.69 × 10^9^
85%:15%	5.53	5.34	2.59	1.15 × 10^−3^	1.45 × 10^−10^	7.92 × 10^6^
80%:20%	8.156	−0.08	0.98	1.99 × 10^−3^	5.89 × 10^−12^	3.38 × 10^8^

**Table 4:** Electrical performance parameters of MgZnO/ZTO-TFTs devices with different thicknesses of ZTO films.

ZTO Film Thickness	μ_sat_ (cm^2^·V^−1^·s^−1^)	V_TH_ (V)	SS (V/dec)	I_on_	I_off_	I_on_/I_off_
45 nm	10.46	2.44	0.02	2.33 × 10^−3^	8.66 × 10^−13^	2.69 × 10^9^
52 nm	16.80	−1.60	0.74	4.18 × 10^−3^	5.48 × 10^−12^	7.63 × 10^8^
60 nm	10.95	−5.47	1.72	3.35 × 10^−3^	1.58 × 10^−11^	2.12 × 10^8^
67 nm	9.66	−7.2	1.99	3.2 × 10^−3^	1.65 × 10^−11^	1.94 × 10^8^

**Table 5:** Stability data.

ΔV_TH_	ZTO-TFTs	MgZnO/ZTO-TFTs
10 V	+4.38 V	+0.58 V
−10 V	−3.32 V	−0.15 V

## Data Availability

The original contributions presented in this study are included in the article. Further inquiries can be directed to the corresponding authors.
